# Topics of Public Interest

**Published:** 1912-04

**Authors:** 


### TOPICS OF PUBLIC INTEREST

THE MONROE COUNTY COMMITTEE ON MENTAL HYGIENE, was organized March 14, 1912, by a public meeting composed both of physicians and laymen.

County Judge John B. M. Stephens was selected to serve as president of the committee, Dr. E. B. Angell, George H. Smith and Dr. Thomas O'Hare as vice-presidents, and Dr. E. L. Hanes as secretary and treasurer.

The committee will endeavor, through public lectures and the dissemination of information through various channels, to teach the essentials of mental hygiene and to provide means for proper and adequate treatment for incipient cases of insanity in special wards at one or more of the city hospitals, and will co-operate with the Central Committee on Mental Hygiene of the State Charities Aid Association, which is a section of the National Charities Aid Society, for the prevention and cure of insanity.

The first lecture meeting was held March 28, with the following program, Dr. Edwin H. Wolcott, Presiding.

"The Causes and Treatment of Mental Diseases," Dr. Smith Ely Jelliffe, New York City. "A Plan of Campaign," Mr. Everett S. Elwood, New York City. "Rochester's Need of a Psychopathic Ward from a Medical Point of View," Dr. Edward L. Hanes. "Rochester's Need of a Psychopatic Ward from a Legal Point of View," Judge J. B. M. Stephens, General discussion.

BACTERIOLOGIC EQUIPMENTS.—Last month, we noted the farming out of the bacteriologic work of Monroe County to a practicing physician. The city of Buffalo started 5 years ago with an appropriation of $600 for equipment. On this annual allowance, the number of tests has increased from 7,660 to 25,160. It is now proposed to double the annual appropriation and to ask something less than $700 for a special appropriation for apparatus. Chicago pays $5,000, Toronto $4,000, Baltimore $3,500, Cleveland $3,000, Des Moines $2,500, Birmingham Ala. $2,000. Major Bissell has done good work with the poor equipment thus far granted him. We need not argue in behalf of his department. Efficiency of this kind was noted and the proper reward suggested many centuries ago. "Well done, good and faithful servant; thou hast been faithful over a few things, I will make thee ruler over many things."

Oxalic Acid Poisoning.—The death of eight infants in the Brooklyn Nursery and Infants' Hospital, by oxalic acid placed in their food by a woman jealous of the nurses and wishing to make them trouble, is a shocking incident. Naturally, the question of her sanity has arisen. In and of itself, the incident does not necessarily imply lack of sanity, but merely ignorance and lack of perspective. The smart jay who daubs a dog's anus with turpentine and shoves the poor beast into a church social; the colored girl seeking a love philtre; the author or dramatist who, as an item in the development of a plot, puts some one to sleep with a safe drug—which the profession would be glad to possess—; the thrifty layman who considers that the "Do not repeat" on a prescription is a form of medical graft; all belong in the same category with the unfortunate woman who has unwittingly dealt death to these little ones. Is there not some way to impress on the laity that drugs are not to be fooled with; that a little knowledge of them is a terribly dangerous thing?

---

The University of Buffalo celebrated "University Day" at the Teck Theatre, on Washington's Birthday. Dr. Roswell Park delivered the address.

---

Mount Morris has recently voted appropriations of $140,000 for water supply; $45,000 for sewerage.

---

The State Board of Regents has rescinded the agreement to endorse Vermont medical licenses, on the ground that the Vermont requirements do not come up to the New York standard. New York, by the way, is very far below the requirement of many western states in regard to general educational requirements, though relatively high on purely technical requirements. According to precedent, the action of the New York regents means also, that New York licenses will not be honored in Vermont. "Reciprocity" is a poor basis for establishing educational and medical attainments. A few years ago the examining board of one of the western states took the dignified and honorable stand that it would recognize the license of any state having an equal or *higher* requirement, and that it would not retaliate upon individuals for the action of a state board, in maintaining a standard which the state in question has not yet attained. Illinois, also, is being cut off the reciprocity list of some states on account of its failure to maintain a high standard.

---

John Morg, died at his home on Indian Creek near Columbia, Ky., February 23, 1912. He was a veteran of the War of 1812.

He immigrated from Germany in 1812 and enlisted, giving his age as 24. He was probably the oldest man in the United States up to his death.

BACTERIOLOGIST, PHILIPPINE SERVICE.

A research bacteriologist will be appointed at a salary of $2,500, subject to the usual perquisites and promotion. Eligibles may also be selected to fill similar vacancies which may arise later. The customary requirements hold as for most government appointments. No formal examination will be held but the selection will be made on investigation of credentials, theses, etc., presented by the applicant. Those interested should apply to the U. S. Civil Service Commission, Washington, D. C., asking for Form B.I.A. 2.

---

Dr. Harvey W. Wiley has resigned, presumably because of friction with the members of the Department of Agriculture who failed to have him ousted. Moral, for the medical profession: Keep out of politics. Dr. Freer of the Philippine service is mentioned as a possible appointee and President Taft has instituted a general inquiry among colleges for available men.

---

Asiatic Cholera.—Naame, a French physician at Tunis, has cured all of a series of 20 cases, using intra-venous injections of adrenalin. The Pasteur Institute endorses his discovery.

---

Small Pox.—Dr. Grover W. Wende discovered a case in a traveling man, in a Buffalo rooming house. The patient came from Grand Rapids but is supposed to have contracted the disease in a hotel in Kalamazoo. Dr. Charles F. Durand of the Quarantine Hospital states that while the patient gives a history of attempted vaccination, it was not successful and no scar is evident. In fact, in an experience of about 70 cases at the hospital, he has never encountered a case that had been successfully vaccinated. Conversely, some fifteen or twenty exposed individuals (mothers accompanying infants or vice versa), immediately vaccinated and remaining at the hospital, have escaped infection. Here are some hard facts for the anti-vaccinationists to explain.

---

The *Medical Union,* one of the oldest medical organizations in Buffalo, held its monthly meeting at the Statler Hotel, February 28, 1912. About 25 members and guests were present. Dr. Clayton M. Brown was the host of the evening. After dinner, Dr. George F. Cott delivered his address as incoming President, emphasizing the practical value, even in achieving professional and scientific advances, of social acquaintance and harmony, as fostered by such societies as the Medical Union.

Dr. George H. Westinghouse then read a brief but complete resume of the laws regarding abortion. The discussion took up various ethical phases of the same subject and several speakers dwelt on the criticisms which had been made of the agencies employed by the Censors of the Society of Erie Co., emphasizing the necessity of using such means as were available. It seemed to be the general experience that, whether convictions were obtained or not, the men who had formerly done much abortion practice had desisted and that patients now had a long hunt before they could find anyone to perform an abortion.

---

### Wild Parsnip Poisoning.

ONE death and a number of cases of acute poisoning are reported from Perry, under date of March 19, due to wild parsnip, dug by one of the children. Wild parsnip is regarded by most botanists as identical with the cultivated vegetable and was probably introduced from Europe, though some consider it as indigenous. Even the cultivated parsnip contains some toxic material, especially in the outer parts of the plant. Opopanax, another species, originally from south-western Asia, has been used in medicine as an antispasmodic but is now practically discarded.

All of the umbelliferae are more or less poisonous, some dangerously so. The compound leaves and globular or disc-like clusters of flowers are so characteristic for the whole family, that it should be easy to warn children against them, by an easily remembered general description.

The mints, though valuable as minor remedies, are not usually regarded as strictly poisonous. However, the writer has seen syncope, apparently of dangerous degree and length of cardiac depression and temporary deafness from a teaspoonful of the oil of peppermint. It is not at all likely that a child would eat enough of any mint to absorb a large dose of any volatile oil. Still, as in the case of the umbelliferae, a fairly accurate general description of the whole family of mints can be set forth as a warning.

Many kinds of mushrooms are poisonous and none of the ordinary distinguishing tests between toxic and non-toxic kinds, are at all reliable in either direction. Children should be taught (and grown persons too) that only those who have given expert attention to the study can distinguish between edible and toxic species, and that the distinction rests on no general rules but on personal acquaintance.

Poison ivy produces much discomfort, occasionally serious lesions though, on the whole, its danger is exaggerated. Children can easily be impressed with its distinction from Virginia creeper. Tell them that the vine with five leaflets, like the fingers of the hand, can safely be taken by the hand but that they must not shake hands with the three-leafleted vines.

Deadly night-shade, can easily be described by the peculiar bright berry and the various shaped leaves. But all the solanaceae, even the potato, tomato and egg plant, are poisonous in their green parts.

We are very rusty on botany—not that we love nature less but ancient remains of man, more, so that the limited time that the busy man can spend in the fields and woods has been otherwise occupied. But among our readers there must be many who can sum up the poisonous plants of this region, classify them and give brief descriptions which physicians generally can pass on to children and grown persons no wiser, in time to protect them against the temptations of growing plants during the coming summer.

